# Chaotic Scattering and Escape Times of Marginally Trapped Ultracold Neutrons

**DOI:** 10.6028/jres.110.055

**Published:** 2005-08-01

**Authors:** K. J. Coakley, J. M. Doyle, S. N. Dzhosyuk, L. Yang, P. R. Huffman

**Affiliations:** National Institute of Standards and Technology, Boulder, CO 80305 USA; Harvard University, Cambridge, MA 02138 USA; North Carolina State University, Raleigh, NC 27695 USA; National Institute of Standards and Technology, Gaithersburg, MD 20899 USA

**Keywords:** chaotic scattering, escape time, Hamiltonian system, magnetic trap, symplectic integration, ultracold neutrons

## Abstract

We compute classical trajectories of Ultracold neutrons (UCNs) in a superconducting Ioffe-type magnetic trap using a symplectic integration method. We find that the computed escape time for a particular set of initial conditions (momentum and position) does not generally stabilize as the time step parameter is reduced unless the escape time is short (less than approximately 10 s). For energy intervals where more than half of the escape times computed for UCN realizations are numerically well determined, we predict the median escape time as a function of the midpoint of the interval.

## 1. Introduction

There are at present several Ultracold neutron (UCN) experiments that seek to measure the neutron lifetime using magnetic confinement techniques (see these proceedings). Experiments are underway and are being proposed that utilize permanent magnets, superconducting magnets, and a combination of the two to create magnetic field geometries that can be used to spatially confine UCNs. Such techniques are advantageous because they minimize (or eliminate) any interactions that a UCN has with the material walls of the container, thus dramatically reducing (or removing) the largest systematic error that has plagued UCN lifetime experiments to date. Reducing the uncertainty of a neutron lifetime measurement beyond roughly a factor of two (relative to the presently accepted value) is a challenge that requires a new approach and/or substantial increases in the number of available UCNs. Since magnetic trapping techniques offer great promise to meet this challenge, they are very popular.

One key systematic effect that arises when using these new magnetic trapping techniques is marginal trapping; neutrons with a total energy greater than the trap depth may be temporarily contained in the trap. For example, a neutron in a circular orbit (with its plane perpendicular to the trap axis) can gradually turn into an elliptical orbit (due to angular momentum nonconservation in an angular dependent field) and thus reach a greater distance from the origin. If the initial energy of this neutron is larger than the trap depth, the neutron can then strike the container wall and be ejected from the trap before it decays. Consequently, the neutron lifetime estimated from data consisting of a mixture of permanently trapped and marginally trapped neutrons will be systematically shorter than the actual lifetime of the neutron.

In this work, as part of the UCN lifetime experiment presently underway at National Institute of Standards and Technology (NIST) [1–3], we present detailed neutron trajectory simulations in an Ioffe-type superconducting magnetic trap. Neutrons with energies higher than the trap depth can escape from the trap. However, the escape of these marginally trapped neutrons is not immediate and we have shown experimentally that it can introduce a systematic error into the measured neutron lifetime. Analytic calculations [4] (which have been experimentally verified) have shown that it is possible to remove practically all of these marginally trapped neutrons from the trap by lowering the depth of the trap for a short time and then raising it again. However, this procedure also reduces the number of nonmarginally, i.e., permanently, trapped neutrons.

To determine the optimal lowered trap depth for removal of practically all of the marginally trapped UCNs, one must understand the escape time distribution of UCNs. In this work, we simulate realizations of UCNs and compute their escape times assuming that the trajectories are classical. The escape time is defined to be the time it takes the UCN to first cross the boundary of a cylindrical detection volume. As a caveat, this model for escape simplifies quantum mechanical aspects of neutron interactions. In a later work, we shall develop a more realistic model for neutron escape that accounts for such effects as described in ref. [5]. For each realization, we compute a trajectory using a symplectic integration algorithm [6–13]. Symplectic integrators were developed for Hamiltonian systems and are generally regarded as superior to Runge-Kutta methods for particle tracking problems such as the one considered in this work. In particular, we implement the optimal fourth order scheme presented in ref [10].

Our primary result is that the computed escape time for a particular set of initial conditions (momentum and position) does not always stabilize. In general, for the cases studied here, computed escape time stabilizes for cases where the neutron promptly escapes (less than approximately 10 s). For longer escape times, stability is generally rare. We interpret the observed instability of the computed escape time as a manifestation of chaotic behavior. For such trajectories, slight differences in computed trajectories due to numerical noise (which depends on the time step size) can be magnified dramatically at advanced times. For more discussion of this sensitivity in related problems, see refs. [8] and [10]. In general, chaotic behavior of computed trajectories for Hamiltonian systems is called chaotic scattering [14,15]. We also present an empirical model to predict the median escape time of UCNs as a function of the midpoint of the particular energy interval. As a caveat, the escape time distribution for UCNs in an actual experiment may also be affected by mechanical vibrations and slight instabilities in the magnetic field. In this work, we do not include these possible effects.

## 2. Physical Model

In the ongoing experiment at NIST, UCNs are produced by inelastic scattering of cold neutrons from a reactor in superfluid ^4^He. By creation of a single phonon in the superfluid, a cold neutron with wavelength near 0.89 nm (speed of approximately 400 m/s) is scattered to a state of near rest. (The mean wavelength of a thermal ensemble of neutrons at 12 K is 0.89 nm (8.9 Å).) The resulting UCNs are confined in a potential field *V* (*x*) produced by the interaction of the magnetic moment of a neutron and a spatially varying magnetic field

**Fig. 1:**
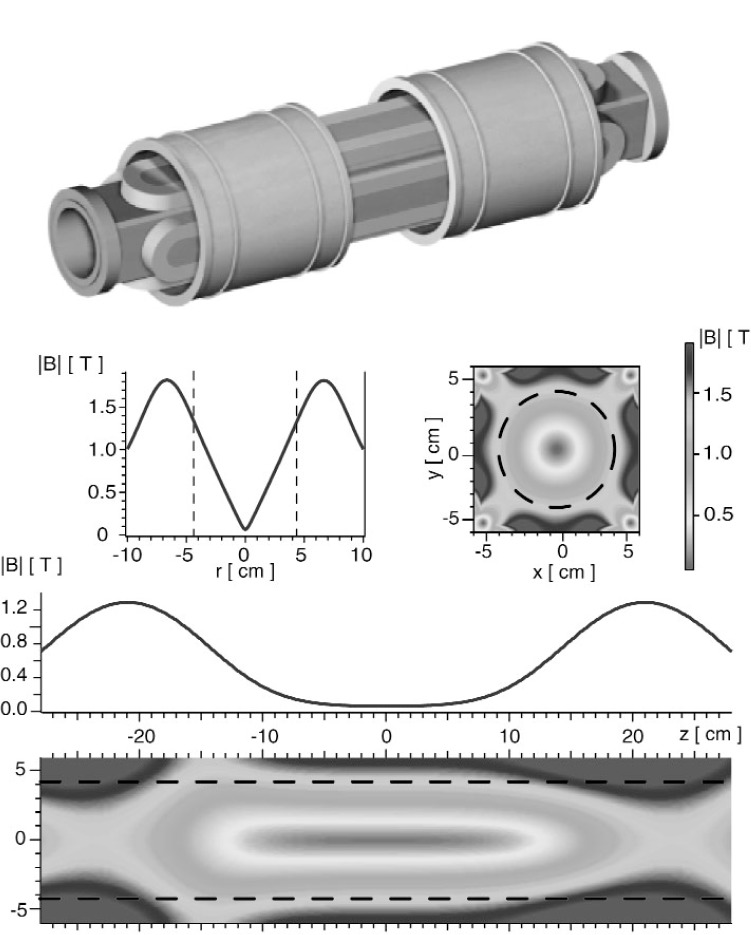
Magnet trap for confining ultracold neutrons [1–3]. A rendering of the magnet coils and form is shown at the top. The two graphs depict the magnitude of the magnetic field as a function of *r* (along *z* = 0) and *z* (along *x* = 0). The two contour plots show the two dimensional field profiles in the *x* – *y* plane (at *z* = 0) and in the *x* – *z* plane (at *y* = 0). The dashed lines denote the physical walls of the trap.

V(x)=μ|B(x)|, (1)

where ***B*** is the magnetic field, |***B***(*x*)| is the magnitude of ***B***, and *µ* is the magnetic moment of the neutron [1,2]. In cylindrical coordinates, the trapping volume is approximately −18 cm ≤ *z* ≤ 18 cm and *r* ≤ 4.3 cm, where $r=\sqrt{x^{2}+y^{2}}$ and is shown in Fig. 1. The maximum of |***B***| on the escape boundary is approximately 2.1 T. The ratio of the minimum to maximum value of |***B***| on the escape boundary is 0.573. This minimum point (|***B***| = 1.2 T) is the trap depth. The total energy of a UCN created at position *x* is *E* = *p*^2^/(2*m*_n_) + *V*(*x*) where *p* is the magnitude of the UCN momentum and *m*_n_ is the mass of the neutron. Here, we focus on the escape times of UCNs with energies less than 1.6 *V*_max_, where *V*_max_ is the maximum potential value on the boundary of the trap. If a UCN had kinetic energy equal to *V*_max_, its speed would be approximately 3.6 m/s. For UCN with low kinetic energies (*p*^2^/(2*m*_n_) ≤ 4*V*_max_), we assume that the UCN velocity direction is uniformly distributed on the unit sphere and that the conditional probability density function of *p* is proportional to *p*^2^. We use this conditional probability density function to simulate realizations of *p* and compute escape times for realizations of UCN initial position and momentum with total energy less than 1.6 *V*_max_.

We assume that the initial position of the UCN is uniformly distributed in the trapping volume. To determine a neutron trajectory based on its initial position and momentum, we solve the classical equations of motion,

p=F(x)=−∇V (2)

$$\dot{x}=p/m_{n},$$ (3)

using an optimal fourth order symplectic integration scheme designed for separable Hamiltonian systems where the kinetic energy is a quadratic function of momentum [10]. A symplectic map *M*(*t*) is a canonical transformation of a point in position-momentum phase space at initial time *t* = 0 to a point in position-momentum phase space at time *t*. That is, (*x*(*t*), ***p***(*t*)) = *M*(*t*)(*x*(0), ***p***(0)). In the optimal fourth order scheme presented in [10], the predicted value of the position and momentum at time t + *∆*, is (*x*_4_, ***p***_4_), where

For *i* = 1,4

$$p_{i}=p_{i-1}+b\left(i\right)F\left(x_{i-1}\right)\Delta$$ (4)

$$x_{i}=x_{i-1}+\frac{a\left(i\right)}{m_{n}}p_{i}\Delta$$ (5)

endfor.

Above, *x*_0_ = *x*(*t*), *p*_0_ = *p*(*t*) and

$$\begin{array}{cc} a\left(1\right)=0.5153528374311229364 & b\left(1\right)=0.1344961992774310892\\ a\left(2\right)=-0.085782019412973646 & b\left(2\right)=-0.2248198030794208058\\ a\left(3\right)=0.4415830236164665242 & b\left(3\right)=0.7563200005156682911\\ a\left(4\right)=0.1288461583653841854 & b\left(4\right)=0.3340036032863214255. \end{array}$$

The coefficients *a*(*i*), *b*(*i*) *i* = 1, …, 4 are numerically determined to minimize an error constant that measures Hamiltonian (total energy) truncation errors. For more details, we refer the reader to [10].

To determine when a neutron crosses the boundary, we use a quadratic interpolation method to estimate the maximum value of *r* and |*z*| for the trajectory based on computed values of *r* and *z* at three successive time steps.

In our numerical approach, we predict |*B*| at arbitrary points in the trapping volume by using a three dimensional tensor-product spline interpolant [16] where the order of the spline is 4 in each direction. We estimate the tensor-product *B*-spline coefficients from values of |*B*| computed on a grid by a numerical code that numerically solves the Biot-Savart law numerically corresponding to the geometry of the solenoid and current bars that produce the magnetic field. The grid spacing in the *x*, *y*, and *z* direction is 0.1 cm, 0.1 cm, and 0.5 cm. The value of the potential and its gradient is evaluated at arbitrary locations given the *B*-spline coefficients.

## 3. Simulation Study

**Fig. 2:**
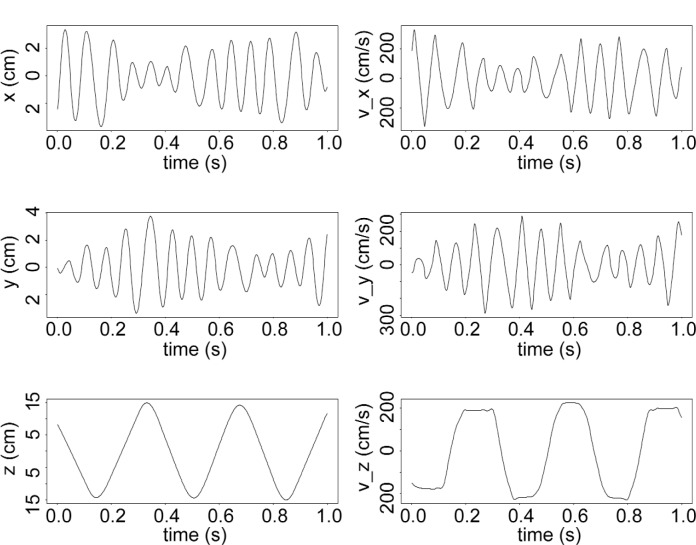
Computed trajectory for CASE A using a fourth order symplectic integration method. The computed escape time is 4.9 s and *E/V*_max_ = 0.651.

**Fig. 3:**
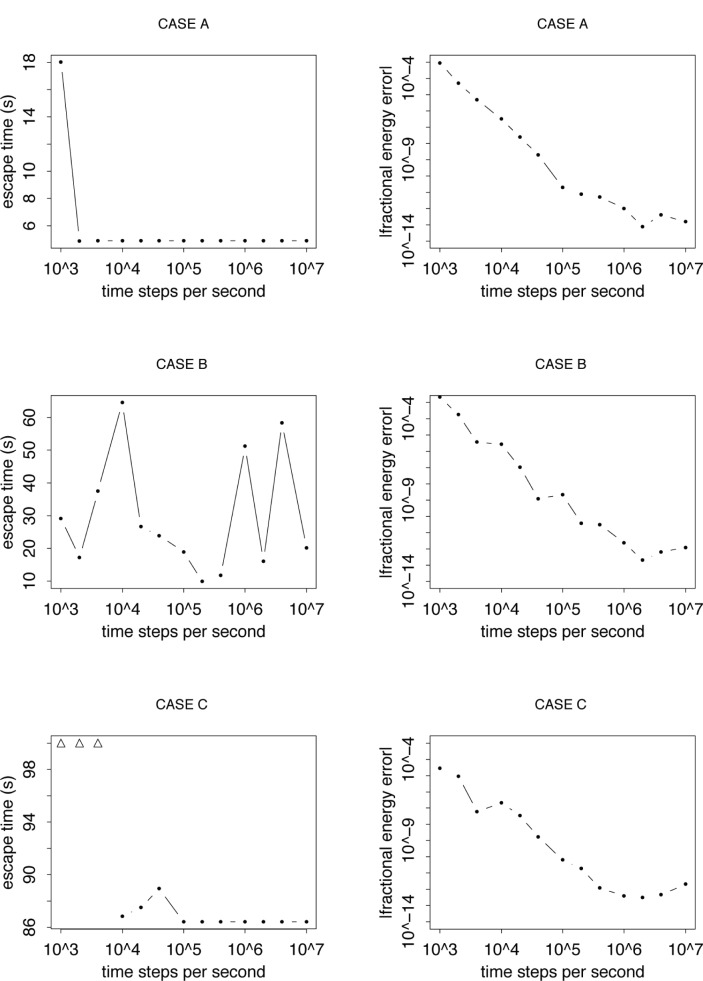
Computed escape times for three cases. For CASES A, B, and C, *E/V*_max_ = 0.651, 0.585, 0.697. The triangular symbols represent cases where the escape time is longer than 100 s.

In Fig. 2, we plot a sample predicted trajectory. For this example, CASE A, the computed escape time stabilizes as the time step in the integration scheme decreases. The fractional absolute error $\left|\left(\hat{E}-E\right)/E\right|$ where *E* is the true energy of the UCN and *Ê* is the predicted energy at escape, generally decreases as the time step decreases (Fig. 3). For other cases, even though the fractional energy at escape is comparable to that observed for CASE A, the computed escape time does not stabilize (see CASE B). For chaotic triatomic systems, Schlier and Seiter remarked that good energy conservation does not ensure that the predicted trajectory is close to the “true” trajectory of interest [11]. Our result is consistent with this remark.

For all cases studied, stability is achieved when the computed escape time is less than approximately 10 s. To illustrate the stability problem for longer escape times, we plot the computed escape time for 25 cases. We halt the trajectory after 100 s if there is no escape. For these cases, we vary the time step from 10^−3^ s to 10^−7^ s. For computed escape times between 10 s and 30 s, stability is sometimes achieved. Beyond 30 s, stability occurs just once for an escape time of 86 s (CASE C).

**Fig. 5:**
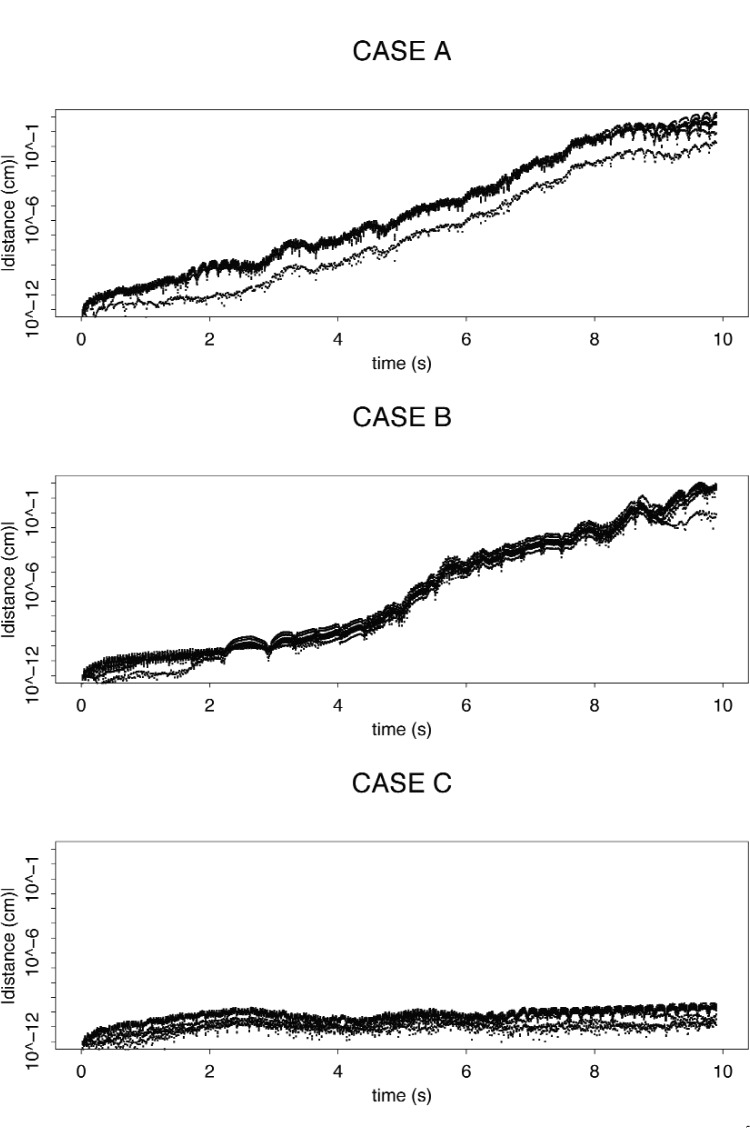
Distance between predicted trajectories with the same initial velocity, but different initial position. 10^5^ time steps per second.

In a sensitivity analysis, we compute the trajectory of interest for the cases A, B, and C. For each case, we predict nine other trajectories with the same initial velocity but slightly different initial positions. For cases A and B, after about 8 s, the distance between the reference trajectory and the other trajectories grew by about ten orders of magnitude (Fig. 5). Thus, it is not a surprise that the computed escape time is unstable for these cases. For CASE C, the distance grew only by about two orders of magnitude. Thus, it is plausible that we can compute a long escape time for CASE C even though we cannot for CASE B.

An open question is whether one can consistently estimate the probability density function of escape times for an ensemble of realizations even though we cannot, in general, determine the escape time for all realizations in the ensemble. This point was raised in refs [8] and [10]. In other words, is the probability density function of computed escape times for an ensemble of trajectories (with random initial positions and velocities) computed for a given time step the same as the probability density function of the true escape times for the ensemble? This is an open question.

**Fig. 6:**
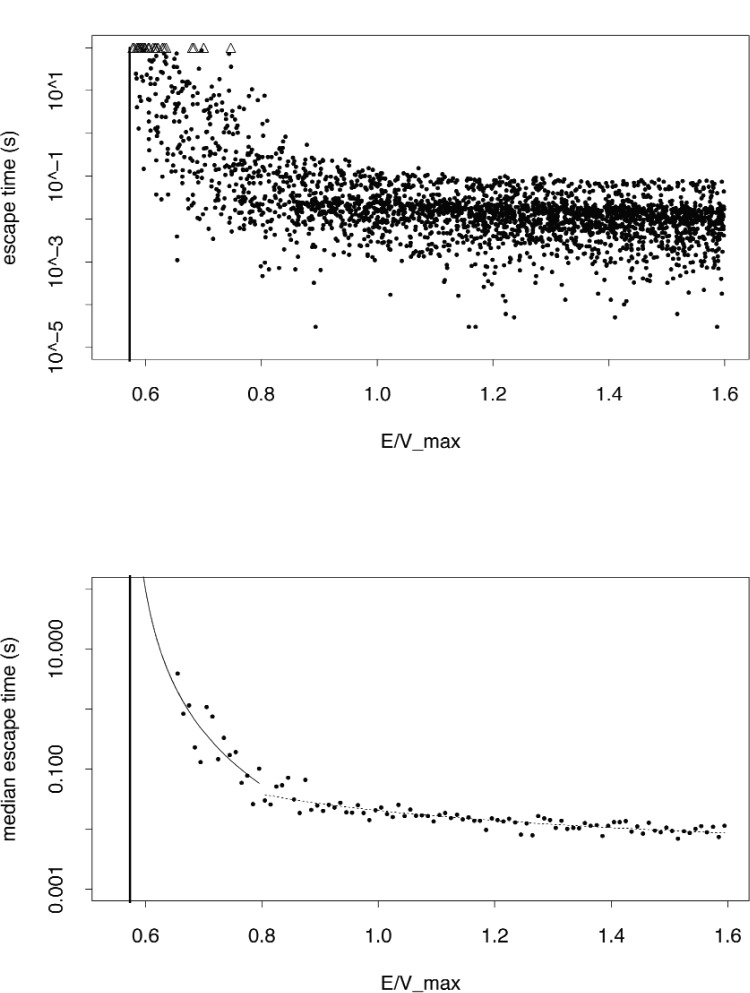
Top: Computed escape times (10^5^ time steps per second) for UCN. Triangular symbols represent cases where the escape time is longer than 100 s. Bottom: We predict the median escape time as a function of the midpoint of energy bins according to Eq. (7).

If more than half of the computed escape times are well determined numerically for given energy interval, the median is well determined for that interval. Thus, we can still extract useful information from the computed escape times even though some may not be well determined. We bin computed escape time data from simulated realizations of UCNs according to energy, and empirically model the logarithm of the median escape time for each bin as a function of the bin midpoint, $\bar{E}$ as follows:

$$\ln\left[MED\left(t_{\text{esc}}/t_{o}\right)\right]\left(\bar{E}\right)=\gamma_{1}+\gamma_{2}\ln\;\left[\frac{\bar{E}-V_{\min}}{V_{\max}}\right]+\varepsilon \left(\bar{E}\right),$$ (6)

where the maximum potential value on the boundary of the trap is *V*_max_ and the minimum potential on the boundary is *V*_min_ = 0.573 *V*_max_ (shown in Fig. 6 as a thick vertical line) *t*_o_ = 1 s and $\varepsilon (\bar{E})$ is the prediction error for the bin with midpoint $\bar{E}$. The energy bins had uniform width of 0.01 *V*_max_. Above 0.655 *V*_max_, the computed median for each bin stabilized as a function of time step (the minimum time step was 10^−5^ s). We determine the model parameters by the method of ordinary least squares and their associated 1-sigma random uncertainties by a nonparametric bootstrap resampling scheme [17–19]. The basic idea of the bootstrap is to generate synthetic data based solely on the observed data without making any distributional assumptions. Our observed data can be represented as *N* data pairs *z*_i_ = (*x*_i_, *y*_i_) *i* = 1, …, *N*, where *x*_i_ is the *i*th energy bin midpoint and and *y*_i_ is the *i*th median escape time. We draw *N* realizations of a random integer that is uniformly distributed between 1 and *N*. Denote this string of random integers as (*j*_1_, *j*_2_, …, *j*_N_). Our bootstrap replication of the observed data is $z_{j_{1}},z_{j_{2}},z_{j_{3}},\ldots ,z_{j_{N}}$. We refit the model to each bootstrap replication of the data and store the associated parameter estimates. The standard deviation of the model parameter estimates computed from the bootstrap data is our estimate of the 1-sigma uncertainty of the model parameter estimated from the observed data. In this study, we simulate 10^4^ bootstrap replications of the data.

For 0.655 $V_{\max}<\bar{E}<0.8V_{\max}$, the estimated parameters are ${\hat{\gamma }}_{1}=-8.16$ and ${\hat{\gamma }}_{2}=-3.53$. The bootstrap estimates of the 1-sigma random uncertainties for these estimates are 1.19 and 0.64 respectively. For *E* > 0.8 *V*_max_, the estimated parameters are ${\hat{\gamma }}_{1}=-4.74$.74 and ${\hat{\gamma }}_{2}=-1.00$. The associated bootstrap estimates of the 1-sigma random uncertainties are respectively 0.04 and 0.09. For clarity, we write the predicted median escape time for the bin with midpoint $\bar{E}$ as

$$\overset{⌢}{ME}D\left(t_{\text{esc}}/t_{o}\right)\left(\bar{E}\right)=\exp\left(\gamma_{1}\right){\left(\frac{\bar{E}-V_{\min}}{V_{\max}}\right)}^{\gamma_{2}}.$$ (7)

## 4. Summary

We modeled the trajectories of UCNs in a magnetic trap classically and predicted the trajectory of the neutron by integrating the laws of motion using a symplectic integration scheme. In our approach, we modeled the potential produced by the spatially varying field in the trap using a spline interpolation scheme. Since this interpolation scheme is an approximate method, the computed escape times may differ from those computed by use of an exact model for the potential. Since each component of the magnetic field in the trap satisfies Laplace’s equation, it might be possible to develop a special function expansion approximation for the potential. As a caveat, the escape time distribution for UCNs in an actual experiment may be affected by mechanical vibrations and slight instabilities in the magnetic field. In this work, we did not include these possible effects.

We defined the escape time of a neutron to be the first time that the classical neutron trajectory crosses the boundary of the trapping volume. The trapping volume boundary is the union of the endcaps and the surface of a cylinder. For all cases considered, we computed numerically stable escape times for trajectories that promptly escaped the trap in about 10 s or less. However, in general, for longer escape times, stability is rare. How to interpret a computed escape for a particular time step for such unstable cases is not clear. Other researchers [8,10] have remarked that one does not expect to compute the escape time for a particular trajectory in a chaotic set in a stable manner. However, they suggested that it may be possible to estimate the probability density function of the escape times for the chaotic set. We are not aware of any proof of this conjecture.

We also predicted the median escape time for simulated UCNs based on escape time data at energies where the median was well determined. The accuracy of this model for lower energies is an open issue. In future work, we plan to study a more realistic model for neutron escape that accounts for additional quantum effects. In this more realistic model, neutrons would escape or scatter off the boundary according to stochastic laws.

## Figures and Tables

**Fig. 4:**
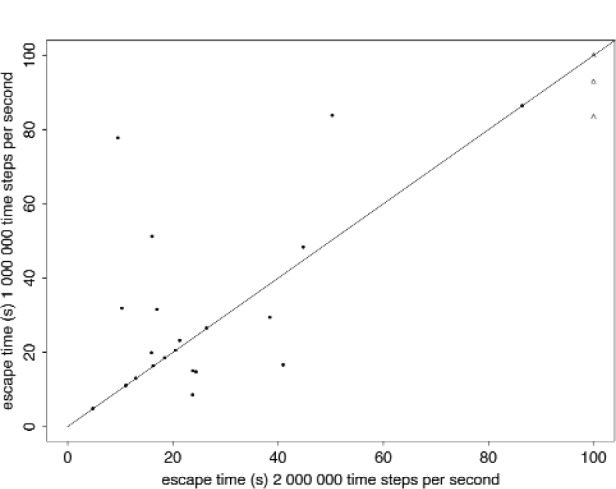
Computed escape times for two time steps for 25 cases. Line of equality drawn. The triangular symbols represent cases where one or both escape times is longer than 100 s.

## References

[1] Huffman PR, Brome CR, Butterworth JS, Coakley KJ, Dewey MS, Dzhosyuk SN, Gilliam DM, Golub R, Greene GL, Habicht K, Lamoreaux SK, Mattoni CE, McKinsey DN, Wietfeldt FE, Doyle JM (2000). Magnetic Trapping of Ultracold Neutrons. Nature.

[2] Brome CR, Butterworth JS, Coakley KJ, Dewey MS, Dzhosyuk SN, Golub R, Greene GL, Habicht K, Huffman PR, Lamoreaux SK, Mattoni CEH, McKinsey DN, Wietfeldt FE, Doyle JM (2001). Magnetic trapping of ultracold neutrons. Phys Rev C.

[3] Huffman PR, Coakley KJ, Dzhosyuk SN, Golub R, Korobkina E, Lamoreaux SK, Mattoni CEH, McKinsey DN, Thompson AK, Yang GL, Yang L, Doyle JM, Abele H, Mund D (2003). Progress Towards Measurement of the Neutron Lifetime Using Magnetically Trapped Ultracold Neutrons.

[4] Brome CR (2000). Magnetic Trapping of Ultracold Neutrons. PhD thesis.

[5] Golub R, Richardson DJ, Lamoreaux SK (1997). Ultra-Cold Neutrons.

[6] Ruth RD (1983). A canonical integration technique. IEEE Transactions on Nuclear Sci.

[7] Forest E, Ruth R (1990). Fourth order symplectic integration. Physica D.

[8] Channel PJ, Scovel C (1990). Symplectic integration of Hamiltonian Systems. Nonlinearity.

[9] Candy J, Rozmus W (1991). A symplectic integration algorithm for separable Hamiltonian functions. J Computational Phys.

[10] McLachlan RI, Atela P (1992). The accuracy of symplectic integrators. Nonlinearity.

[11] Schlier Ch, Seiter A (2000). High-order symplectic integration: an assessment. Computer Physics Communications.

[12] Wu YK, Forest E, Robin DS (2003). Explicit symplectic integrator of s-dependent static magnetic field. Phys RevE.

[13] Levichev EB, Piminov PA (2002). Symplectic integrator for particle tracking in complex magnetic field.

[14] Bleher S, Grebogi C, Ott E, Brown R (1988). Fractal boundaries for exit in Hamiltonian dynamics. Phys Rev A.

[15] Breyman W, Kovacs Z, Tel T (1994). Chaotic scattering in the presence of an external magnetic field. Phys Rev E.

[16] de Boor C (1978). A practical guide to splines.

[17] Efron B, Tibshirani RJ (1991). Statistical data analysis in the computer age. Science.

[18] Efron B, Tibshirani RJ (1993). An introduction to the bootstrap.

[19] Chernick MR (1999). Bootstrap methods a practitioners guide.

